# Quasi-experimental evaluation of a digital occupational health management system on presenteeism and work efficiency among healthcare workers

**DOI:** 10.3389/fpubh.2026.1827192

**Published:** 2026-06-04

**Authors:** Feng Qiao, Hui Zhou, Wei Ling

**Affiliations:** 1 Taizhou Fourth People's Hospital, Taizhou, Jiangsu, China; 2 Taizhou Hospital of Traditional Chinese Medicine, Taizhou, Jiangsu, China; 3 The Affiliated Taizhou People's Hospital of Nanjing Medical University, Taizhou, Jiangsu, China

**Keywords:** difference-in-differences, digital intervention, healthcare workers, Job Demands–Resources model, occupational health management, organizational support, presenteeism, wellbeing

## Abstract

**Background:**

Presenteeism, defined as working while unwell, is a common occupational health issue that compromises healthcare worker wellbeing, work efficiency, and patient safety. High workload, insufficient organizational support, and limited systematic health management may exacerbate presenteeism in hospital settings. This study aimed to develop and evaluate a Digital Occupational Health Management System (OHMS), grounded in the Job Demands–Resources model, to reduce presenteeism and improve work-related outcomes among healthcare workers.

**Methods:**

A quasi-experimental pre–post study with a matched contemporaneous control group was conducted among healthcare workers at Taizhou Fourth People's Hospital, Jiangsu, China. The OHMS was implemented over 12 months, with the primary effectiveness assessment performed at 6 months and the subsequent 6 months used for continued monitoring, implementation maintenance, and system optimization. A total of 300 healthcare workers were included in the OHMS intervention group, and 300 contemporaneous non-intervention controls were selected using the same eligibility criteria after propensity score matching. The primary outcome was presenteeism, assessed using the Stanford Presenteeism Scale-6. Secondary outcomes included psychological wellbeing, emotional exhaustion, and objective work performance indicators. Difference-in-differences analysis was used as the primary analytical approach.

**Results:**

After OHMS deployment, 286 of 300 participants completed system registration, with a registration rate of 95.3%. The average monthly active usage rate during the 6-month primary evaluation period was 82.4%. Compared with the matched control group, the intervention group showed greater reductions in presenteeism, emotional exhaustion, sick leave days, overtime hours, and absenteeism rate, as well as greater improvements in psychological wellbeing and work efficiency. Multiple regression showed that workload was positively associated with presenteeism, whereas organizational support, system usage frequency, and wellbeing were negatively associated with presenteeism. Structural equation modeling supported a pathway in which workload increased presenteeism directly and indirectly through reduced wellbeing.

**Conclusion:**

The OHMS intervention was associated with reduced presenteeism, improved psychological wellbeing, and better work-related performance. Longer follow-up and multicenter validation are warranted.

## 1 Introduction

The occupational health and wellbeing of healthcare workers are critical determinants of healthcare quality, patient safety, and organizational performance. In clinical settings, “presenteeism”—working despite suboptimal physical or mental health—has become increasingly prevalent and raises significant concerns (1–3). Presenteeism compromises employee health. It also reduces attention, impairs decision-making, and increases error rates. These effects directly threaten patient safety. Evidence suggests that productivity losses and economic costs associated with presenteeism are typically two to three times greater than those from absenteeism, with a more profound impact on organizational effectiveness (4, 5). Systematic reviews indicate that presenteeism is particularly pronounced in healthcare, especially among nurses (6–8).

In China, healthcare workers face high-intensity, fast-paced workloads, night shifts, emotional labor, and staffing shortages. Combined with a cultural emphasis on dedication and self-sacrifice, these factors contribute to widespread presenteeism in hospitals (9–11). Although international studies have addressed the adverse effects of presenteeism, most research has focused on manufacturing, education, and administrative sectors, leaving a gap in empirical evidence and effective interventions for healthcare systems, particularly in resource-constrained Chinese hospitals (1, 2). Data from Sichuan indicate that approximately 65.5% of nurses experience high levels of presenteeism, with psychological disengagement negatively associated with its prevalence (1). Workload, emotional labor, and insufficient social support are key determinants (2), and ICU-based studies further highlight the role of occupational self-efficacy and social support in moderating presenteeism tendencies (3, 12–14).

Presenteeism has been linked to musculoskeletal pain, depression, sleep disorders, and mental fatigue (15), as well as diminished nursing quality, increased patient safety risks, and occupational burnout (11). Organizational interventions, including flexible scheduling and psychosocial support, have been shown to mitigate presenteeism effectively (16). However, occupational health management in Chinese hospitals remains largely reactive and static, focusing primarily on routine physical examinations or isolated psychological assessments. Few initiatives integrate multidimensional physiological–psychological–behavioral data or employ dynamic risk monitoring (16–18).

To address these limitations, this study developed an Occupational Health Management System (OHMS) to transition from static, single-dimensional management to a dynamic, multidimensional, closed-loop approach. The objectives were to: (1) develop a digital platform that integrates automated multi-source health data, intelligent risk stratification, and individualized feedback; (2) empirically evaluate its effectiveness in reducing presenteeism, enhancing wellbeing, and improving work performance using the Job Demands–Resources (JD–*R*) model and Health Belief Model (HBM) (13, 14, 19), and (3) explore feasible implementation pathways and organizational mechanisms for digital health management in hospital settings.

Policy support and resource allocation are essential for scaling OHMS to broader public health contexts. Government subsidies and strategic resource deployment can facilitate adoption in primary care and resource-limited settings, while health insurers may provide additional incentives to enhance engagement and sustainability. Ultimately, OHMS has the potential to improve healthcare worker health and productivity and serve as a valuable tool for enhancing public health management and mitigating presenteeism, offering both practical and academic significance.

## 2 Methods

### 2.1 Study design

This study employed a quasi-experimental pre–post design with a matched contemporaneous control framework to evaluate the effectiveness of the Occupational Health Management System (OHMS) among healthcare workers. The study was conducted at Taizhou Fourth People's Hospital, Jiangsu, China, over a 12-month OHMS implementation period. Taizhou Fourth People's Hospital is a regional public hospital providing outpatient, inpatient, emergency, medical technology, and administrative services, allowing the inclusion of healthcare workers from multiple occupational categories and clinical settings. The primary effectiveness endpoint was assessed at 6 months after OHMS implementation, while the remaining 6 months were used for continued monitoring, implementation maintenance, and system optimization. A total of 300 healthcare workers were included in the OHMS intervention group. During the same study period, an eligible contemporaneous non-intervention control pool was identified from comparable departments using the same inclusion and exclusion criteria. Participants in the intervention group received the OHMS-based intervention, whereas individuals in the eligible contemporaneous control pool received routine occupational health management without OHMS. Outcome measures were collected at baseline and at the 6-month assessment to ensure temporal comparability. After 1:1 propensity score matching, all 300 intervention participants were successfully matched with 300 contemporaneous controls selected from the eligible control pool, resulting in 300 matched pairs for the primary difference-in-differences analysis.

Historical control data were used only for supplementary sensitivity analysis and were not included as the primary comparator. Therefore, all primary effectiveness conclusions were based on the matched contemporaneous control analysis, whereas historical controls were used only to examine the directional consistency of the findings and were not used for causal inference. This supplementary analysis was performed to examine whether the direction of intervention effects remained consistent when compared with pre-implementation occupational health records.

Given the non-randomized nature of the study, propensity score matching was applied to reduce selection bias and baseline confounding. Propensity scores were estimated using a logistic regression model including age, gender, years of service, and department type. A 1:1 nearest-neighbor matching algorithm with a caliper width of 0.2 of the standard deviation of the logit of the propensity score was used without replacement. Covariate balance after matching was assessed using standardized mean differences, with values < 0.10 indicating acceptable balance.

The study was conducted in three sequential phases. The first phase was system development, during which the OHMS platform was designed based on needs assessment and expert consultation. The second phase included baseline assessment and intervention implementation, during which participants in the intervention group received multi-level interventions guided by OHMS, while the matched contemporaneous control group continued to receive routine occupational health management. The third phase was the 6-month post-intervention evaluation, during which outcome measures were reassessed in both groups and qualitative data were collected through semi-structured interviews to enhance interpretation of the quantitative findings.

Difference-in-differences analysis was pre-specified as the primary analytical approach to estimate the net effect of the OHMS intervention by comparing baseline-to-6-month changes between the intervention group and the matched contemporaneous control group. Historical control data were analyzed only as supplementary sensitivity evidence and were not used in the primary DID model. The same pre-defined matching strategy and analytical framework were applied consistently to improve internal validity, methodological coherence, and reproducibility. All matching procedures were performed using *R* software with a fixed random seed to ensure reproducibility.

### 2.2 Study population and sample

Participants included healthcare workers, including physicians, nurses, medical technicians, and administrative staff, at Taizhou Fourth People's Hospital. For the OHMS intervention group, 320 healthcare workers were invited to participate, and 300 valid responses were obtained, yielding a response rate of 93.8%.

During the same study period, 438 healthcare workers from comparable departments were screened for contemporaneous control eligibility using the same inclusion and exclusion criteria. Among them, 18 were excluded, including 10 with incomplete baseline or follow-up data, 5 who were reassigned or transferred to another department or institution during follow-up, and 3 who were on long-term sick leave during the study period. Therefore, an eligible contemporaneous non-intervention control pool of 420 healthcare workers was established. Individuals in this eligible control pool did not receive the OHMS-based intervention and continued to receive routine occupational health management. After 1:1 propensity score matching, 300 controls were selected from the eligible contemporaneous control pool and matched to the 300 OHMS intervention participants, resulting in 300 matched pairs for the primary difference-in-differences analysis.

Because OHMS was implemented as a hospital-wide occupational health management program, the sample size was determined by the number of eligible healthcare workers available during the study period rather than by an *a priori* power calculation. This pragmatic sampling approach was consistent with the real-world implementation nature of the study. Nevertheless, the final matched sample of 300 pairs provided sufficient precision for estimating changes in the primary outcome and allowed adjustment for key demographic and occupational covariates in the DID model.

The historical control group was selected from healthcare workers who met the same eligibility criteria before OHMS implementation and had complete occupational health management records. Historical control data were collected from March 2023 to March 2024 and were used only for supplementary sensitivity analysis. Historical controls were not included as the primary comparator in the main difference-in-differences analysis. The purpose of this supplementary analysis was to assess whether the observed intervention effects were directionally consistent with pre-implementation occupational health records.

The inclusion criteria were as follows: (1) active employment at the hospital for at least 6 months; (2) physician, nurse, medical technician, or administrative staff position; (3) availability of baseline and post-intervention assessment data; (4) no severe physical or mental condition that substantially impaired work participation; and (5) voluntary participation with signed informed consent.

The exclusion criteria were as follows: (1) long-term sick leave during the study period; (2) reassignment or transfer to another institution during follow-up; (3) refusal to participate or withdrawal of consent; and (4) incomplete key questionnaire or occupational health data.

Stratified sampling was performed according to department type, including clinical departments, nursing units, medical technology departments, and administrative departments, to ensure representativeness across occupational categories. The same eligibility criteria and matching variables were applied to the contemporaneous control pool and historical control group where applicable.

### 2.3 Occupational health management system (OHMS)

#### 2.3.1 System architecture and core modules

The OHMS system, co-developed by the hospital IT department and research team, features a modular and scalable architecture compliant with national hospital informatization standards (20). Security protocols followed relevant network and healthcare data protection regulations (21, 22).

The system consists of four core modules: (1) Health data collection: interfaces with HIS, LIS, and RIS systems to monitor psychological health and occupational wellbeing via automated survey prompts; (2) Risk identification and early warning: a multidimensional risk scoring model based on XGBoost was used to support individualized risk stratification, with alert thresholds dynamically defined according to department- and role-specific percentile distributions; (3) Intervention feedback and performance assessment: generates personalized intervention plans with a “health points” feedback system to enhance adherence; (4) System security and access control: ensures data privacy, stability, and regulatory compliance.

Workflow: Data collection → Risk identification → Personalized intervention → Effect evaluation → Management feedback, forming a sustainable digital closed-loop system. In low-resource environments, OHMS supports offline data collection and simplified functionality to maintain operation under limited infrastructure.

#### 2.3.2 Objective behavioral and performance metrics

To reduce self-report bias and improve the objectivity of intervention evaluation, OHMS integrated anonymized behavioral, physiological, and performance-related metrics from multiple data sources.

Work efficiency was assessed using operational log indicators, including outpatient record completion, prescription processing time, and laboratory report response time, which were used to reflect task execution and cognitive performance.

Work quality was evaluated using indicators such as medical near-miss reporting rates and record completeness, which were used to assess attention, accuracy, and work-related performance quality.

Physiological stress was monitored in high-risk staff members using wearable devices, including resting heart rate, heart rate variability, and sleep quality, to provide supplementary information on physiological strain and recovery status.

During system development, an internal XGBoost-based risk stratification algorithm was embedded in OHMS to support individualized alerts (17, 18). Because the present study focused on evaluating the intervention effect rather than developing a prediction model, algorithmic model performance was not treated as a primary study endpoint. Risk thresholds were dynamically set by percentile based on department and occupational role. Data standardization and FHIR-based middleware were used to support secure, real-time integration of heterogeneous HIS/LIS data. End-to-end encryption and de-identification procedures were implemented to protect personal privacy.

### 2.4 Intervention design

The OHMS intervention was implemented over a 12-month period. All primary outcome analyses were based on changes from baseline to the 6-month assessment. During this initial 6-month phase, participants received structured OHMS-based interventions, including individualized feedback, risk alerts, health education, psychological support, workload-related recommendations, and organizational support measures. The subsequent 6 months were used for continuous monitoring, intervention maintenance, and system optimization, but were not included in the primary effectiveness analysis. Interventions were implemented at three levels:

Individual: Personalized risk feedback, tailored interventions, including mental health courses and exercise prescriptions, and positive reinforcement via “health points.”

Team: manager training and team health promotion activities, such as group exercises and wellness workshops, to foster a supportive environment.

Organizational: Policy optimization, including shift scheduling, psychological support, and health culture initiatives to reduce presenteeism. The stratified OHMS intervention design and corresponding implementation objectives are detailed in Table 1.

**Table 1:** Stratified OHMS intervention design and implementation objectives.

Level	Intervention content	Implementation method	Objective
Individual	Health assessment report delivery, psychological counseling and emotional support, exercise and sleep management	Combination of automatic push via OHMS app and face-to-face counseling	Reduce SPS-6 scores and improve WHO-5 wellbeing
Team	Optimized shift scheduling, peer support groups, emotional release activities	Organized by department heads and nurse managers	Reduce perceived workload and team fatigue index
Organizational	Establish a health-supportive culture, implement presenteeism monitoring, integrate health indicators into performance evaluation	Led by hospital management and incorporated into annual objectives	Enhance overall satisfaction and organizational belonging

OHMS refers to the occupational health management system.

The intervention framework is based on behavioral science and organizational management principles. The implementation period was 12 months.

Monthly dynamic evaluations were conducted for at-risk individuals; if an individual remained at high risk for two consecutive assessments, the Occupational Health Management Committee conducted targeted interviews and implemented comprehensive interventions.

Monthly dynamic assessments were conducted during the implementation period. For individuals identified as high risk, additional interventions were triggered, including targeted interviews, psychological counseling, workload adjustment recommendations, and comprehensive support from the Occupational Health Management committee: primary outcomes were assessed at 6 months after OHMS implementation. The later monitoring phase was used to maintain intervention adherence, refine system functions, and support long-term implementation, rather than to estimate the primary intervention effect.

### 2.5 Data collection and measurement instruments

Data collection followed a pre-defined framework based on the study objectives and the Job Demands–Resources (JD–*R*) model, with variables categorized *a priori* into primary outcomes, secondary outcomes, independent predictors, covariates, and qualitative contextual measures to enhance methodological clarity and reproducibility.

Primary outcome: presenteeism was assessed using the Stanford Presenteeism Scale (SPS-6) (23), a validated instrument measuring productivity loss associated with working while experiencing health-related problems. Higher SPS-6 scores indicate greater presenteeism severity and reduced work functioning.

Secondary outcomes: secondary outcomes included psychological wellbeing, occupational burnout, and objective work performance indicators. Wellbeing was measured using the WHO-5 Wellbeing Index (24), with scores ≤ 13 indicating reduced wellbeing. Occupational burnout was assessed using the Maslach Burnout Inventory (MBI) (25), particularly focusing on emotional exhaustion dimensions. Objective work performance indicators included medical error rates, outpatient record completion, prescription processing time, absenteeism rate, overtime hours, and sick leave days.

Independent variables: key predictors were selected based on the JD–*R* theoretical framework and included workload, perceived organizational support, and system usage frequency. Workload was assessed using the NASA Task Load Index (NASA-TLX) (26) and supplemented by objective workload indicators, including weekly working hours, number of night shifts, and consecutive working days. Perceived organizational support was measured using a standardized validated perceived support scale adapted for healthcare workers, with higher scores indicating stronger perceived institutional support. System usage frequency was derived from OHMS operational logs, including login frequency, assessment completion rate, and task engagement.

Covariates: potential confounding variables included age, gender, years of service, and department type. These variables were pre-defined and adjusted for in multivariable analyses to reduce confounding bias.

Physiological and work-related indicators: to complement psychological and behavioral measures, physiological indicators—including systolic and diastolic blood pressure—were extracted from occupational health records before intervention and at follow-up. Work-related indicators included monthly workload hours, number of night shifts, consecutive working days, and near-miss incidents, allowing multidimensional occupational health assessment.

Objective performance metrics: to improve measurement objectivity and reduce sole reliance on self-reported data, SPS-6 scores were correlated with objective performance indicators. Medical error rates were obtained from the hospital quality management system, outpatient record completion reflected operational efficiency, and prescription processing time represented task execution speed under clinical workload conditions. Specifically, outpatient record completion was defined as the number of completed outpatient medical records per month. Prescription processing time was calculated as the average time interval, in minutes, from prescription generation to completion of pharmacist review. Medical error and near-miss indicators were extracted from the hospital quality management reporting system and were standardized according to monthly workload volume where applicable. Sick leave days, absenteeism rate, overtime hours, and work efficiency indicators were obtained from hospital administrative and attendance records.

Digital literacy and technology acceptance: digital literacy and technology acceptance were assessed using items adapted from the Unified Theory of Acceptance and Use of Technology (UTAUT) framework and were used to explore potential factors associated with OHMS engagement.

Qualitative data collection: semi-structured interviews were conducted with healthcare workers across different occupational and risk categories to explore behavioral drivers, system usability, and perceptions of occupational health culture. Participants for qualitative interviews were purposively sampled from low-, medium-, and high-risk groups and from different occupational roles, including physicians, nurses, medical technicians, and administrative staff. Interviews were conducted using pre-defined thematic guides and continued until no substantially new themes emerged. The qualitative findings were analyzed to contextualize and complement the quantitative results.

Validity assessment: to examine criterion-related validity, Spearman correlation analyses were conducted between SPS-6 scores and objective work performance indicators. Higher SPS-6 scores were hypothesized to correlate negatively with efficiency-related indicators and positively with medical error rates, thereby supporting the validity of presenteeism measurement in this occupational setting.

### 2.6 Statistical analysis

All statistical analyses were conducted using SPSS version 26.0 (IBM Corp., Armonk, NY, USA), *R* version 4.2.1 (*R* Foundation for Statistical Computing, Vienna, Austria), AMOS version 27.0 (IBM Corp.), and NVivo version 12. A two-sided *P*-value < 0.05 was considered statistically significant.

The analytical framework was pre-defined and hierarchically structured to improve methodological transparency and reproducibility. The primary hypothesis focused on evaluating the effectiveness of the OHMS intervention in reducing presenteeism and improving occupational wellbeing. Difference-in-differences (DID) analysis was designated as the primary analytical approach to estimate intervention effects, while secondary analyses—including multivariable regression, structural equation modeling (SEM), correlation analysis, and qualitative thematic analysis—were used to identify associated predictors, explore theoretical pathways, and validate measurement consistency.

### 2.7 Descriptive analysis and baseline comparisons

Continuous variables were expressed as mean ± standard deviation (SD), and categorical variables were summarized as frequencies and percentages. Between-group baseline comparisons were performed using independent-samples *t*-tests or Mann–Whitney *U*-tests for continuous variables, depending on distributional assumptions, and χ^2^ tests for categorical variables. For matched samples, paired comparisons were performed when appropriate. Covariate balance after propensity score matching (PSM) was assessed using standardized mean differences (SMD), with SMD < 0.1 considered indicative of acceptable balance.

Primary analysis: difference-in-differences (DID): DID analysis was used as the primary method to evaluate the net effect of the OHMS intervention by comparing baseline-to-6-month changes between the intervention group and the matched contemporaneous control group. Regression models included group, time, and group × time interaction terms, with the interaction term representing the estimated intervention effect. The DID model was specified as follows: Outcome = β0 + β1Group + β_2_Time + β3(Group × Time) + β4Covariates + ε, where Group indicated the OHMS intervention group vs. the matched contemporaneous control group, Time indicated baseline vs. the 6-month assessment, and β3 represented the estimated net intervention effect. Covariates included age, gender, years of service, and department type to reduce residual confounding.

Propensity score matching was implemented before the DID analysis to improve baseline comparability between the intervention and contemporaneous control groups. A pre-defined 1:1 nearest-neighbor matching procedure without replacement was used, with a caliper width of 0.2 of the standard deviation of the logit of the propensity score. Covariate balance was evaluated using standardized mean differences before and after matching, and an SMD < 0.10 was considered indicative of acceptable balance. All post-matching SMDs were below 0.10, supporting adequate baseline comparability between the two groups.

Because formal verification of the parallel trends assumption was limited by the absence of multiple pre-intervention measurements, baseline comparability was strengthened through PSM and assessment of pre-intervention outcome equivalence. Although this approach improved the plausibility of the DID design, the parallel trends assumption could not be fully confirmed. Therefore, the DID estimates should be interpreted as adjusted quasi-experimental intervention effects rather than definitive causal effects.

### 2.8 Within-group pre–post comparisons

Within-group changes in psychological indicators, occupational metrics, and objective performance outcomes were assessed using paired *t*-tests for normally distributed variables or Wilcoxon signed-rank tests for non-normally distributed variables.

### 2.9 Multivariable regression analysis

Multiple linear regression models were constructed with SPS-6 score (23) as the dependent variable to identify independent predictors of presenteeism. Independent variables included workload, perceived organizational support, system usage frequency, and WHO-5 wellbeing score (24), while age, gender, years of service, and department type were included as covariates. Regression assumptions were evaluated through examination of normality, homoscedasticity, and multicollinearity. Variance inflation factors (VIF) < 5 were considered indicative of acceptable collinearity.

### 2.10 Structural equation modeling (SEM)

SEM was performed to test hypothesized mediation and moderation pathways derived from the Job Demands–Resources (JD–*R*) framework (13, 14). A two-step modeling approach was used, including confirmatory factor analysis (CFA) for measurement validation followed by structural model estimation using maximum likelihood methods (27). Model adequacy was assessed using multiple fit indices, including χ^2^/df (< 3.0), root mean square error of approximation (RMSEA < 0.08), comparative fit index (CFI >0.90), Tucker–Lewis index (TLI >0.90), and standardized root mean square residual (SRMR < 0.08). Indirect effects were estimated using bootstrap resampling (5,000 iterations) with bias-corrected 95% confidence intervals.

### 2.11 Correlation and validity analysis

Spearman correlation analysis was used to evaluate associations between SPS-6 scores (23) and objective work performance indicators, including medical error rates, outpatient record completion, and prescription processing time, to assess criterion-related validity.

### 2.12 Missing data handling and sensitivity analysis

Missing data rates were < 5% and were handled using complete-case analysis. Sensitivity analyses were conducted using complete-case datasets and alternative covariate-adjusted DID models to assess the robustness of the primary findings. Additional exploratory analyses were performed to examine whether non-active user status was associated with SPS-6 scores and objective performance indicators, in order to assess potential adherence-related bias.

### 2.13 Qualitative data analysis

Qualitative interview data were analyzed using thematic analysis with NVivo 12. Open coding, axial coding, and thematic categorization were performed independently by two researchers. Inter-rater reliability was assessed using Cohen's kappa coefficient to ensure coding consistency and interpretive rigor.

## 3 Results

### 3.1 Participant characteristics and OHMS usage

Before propensity score matching, 300 healthcare workers were included in the OHMS intervention group, and 420 eligible healthcare workers were included in the contemporaneous non-intervention control pool. After 1:1 nearest-neighbor matching, all intervention participants were successfully matched with 300 contemporaneous controls selected from this eligible control pool, resulting in 300 matched pairs for the primary difference-in-differences analysis. Baseline characteristics were well balanced after matching, with all standardized mean differences below 0.10 (Supplementary Table S1).

In the OHMS intervention group, a total of 300 healthcare workers were included, comprising 98 physicians, 156 nurses, and 46 medical technicians or administrative staff. Female participants accounted for 68.7%, with a mean age of 35.2 ± 6.4 years and an average years of service of 9.7 ± 5.3 years.

Following deployment of the OHMS, 286 of 300 participants completed system registration, corresponding to a registration rate of 95.3%. Based on the pre-defined active-user criterion, 247 participants were classified as active users, whereas 53 participants were classified as non-active users. Active users were defined as participants who logged in at least three times per month and completed at least one assessment or health-related task. The average monthly active usage rate within 6 months of system launch was therefore 82.4%.

High-risk alerts were triggered for 52 participants, accounting for 17.3% of the intervention group. These included 28 cases of psychological risk, 17 cases of workload-related risk, and 7 cases of physiological health abnormalities. Among participants who received individualized interventions, 47 completed the assigned intervention tasks, yielding an intervention completion rate of 90.2%.

Within the OHMS intervention group, participants were further stratified into low-, medium-, and high-risk groups according to the OHMS risk assessment algorithm. As shown in Table 2, 164 participants were classified as low risk, 84 as medium risk, and 52 as high risk. Higher risk levels were associated with older age, longer years of service, longer weekly working hours, and more frequent night shifts, whereas sex distribution did not differ significantly across risk groups.

**Table 2:** Risk level distribution and baseline characteristics within the OHMS Intervention group (*n* = 300).

Variable	Low-risk group (*n* = 164)	Medium-risk group (*n* = 84)	High-risk group (*n* = 52)	χ^2^/F	*P*-value
Female, %	65.2	70.3	76.9	2.11	0.18
Age, years	34.1 ± 5.9	35.8 ± 6.7	37.6 ± 6.1	3.48	0.032
Years of service	8.3 ± 4.8	9.9 ± 5.4	11.2 ± 5.9	4.27	0.018
Average weekly working hours	48.2 ± 6.5	53.4 ± 7.1	58.6 ± 8.3	16.27	< 0.001
Night shifts per month	4.1 ± 2.6	6.8 ± 3.4	8.9 ± 4.2	19.84	< 0.001

This table describes risk stratification within the OHMS intervention group only.

The contemporaneous non-intervention control group was used for the matched difference-in-differences analysis and is summarized separately in the baseline balance table.

Continuous variables are presented as mean ± standard deviation, and categorical variables are presented as percentages. Between-group comparisons were performed using one-way analysis of variance or χ^2^ tests, as appropriate.

OHMS, occupational health management system.

These findings indicate that the OHMS risk stratification was closely related to workload-related characteristics, supporting the relevance of work demand indicators in identifying healthcare workers at higher occupational health risk. Further analysis of the 53 non-active users showed no significant differences in age or years of service compared with active users (*P* > 0.05). However, departmental distribution differed significantly, with a relatively higher proportion of non-active users in administrative and logistical units. Qualitative feedback indicated that the main reasons for non-activity were heavy workload, limited familiarity with digital tools, and hesitation in using new digital health management systems.

#### 3.1.1 Characteristics of non-active users and potential bias

Six months after the OHMS deployment, non-active users—defined as participants who logged in fewer than three times per month or did not complete any assessment tasks—were further analyzed. Stratification by role revealed a higher proportion of non-active users in administrative and logistical departments, whereas clinical and medical-technical departments exhibited significantly higher activity rates than the overall average (χ^2^ = 12.34, *P* = 0.002).

Further analysis indicated that digital literacy scores (measured using the UTAUT scale) were positively correlated with system activity (*r* = 0.41, *P* < 0.01), suggesting that technology acceptance influences adherence to system use. Multiple regression analysis showed that non-active status exerted a modest bias on SPS-6 scores and objective performance metrics (outpatient record completion, medical error rate; β = 0.12–0.18, *P* < 0.05), indicating that the characteristics of non-active users may affect the overall evaluation of intervention effectiveness.

### 3.2 Pre- and post-intervention changes in key indicators

Table 3 presents changes in major psychological, behavioral, and management performance indicators from baseline to the 6-month assessment in the OHMS intervention group and the matched contemporaneous control group. Difference-in-differences (DID) analyses showed that the intervention group exhibited significantly greater improvements across multiple key measures compared with the matched control group.

**Table 3:** Baseline and 6-month changes in psychological, behavioral, and management performance indicators in the OHMS intervention group and matched contemporaneous control group.

Indicator category	Specific measure	Pre-intervention experimental, mean ±SD	Post-intervention experimental, mean ±SD	Pre-intervention control, mean ±SD	Post-intervention control, mean ±SD	DID estimate	*P*-value
Psychological and behavioral	SPS-6 score	20.8 ± 6.1	15.3 ± 5.2	21.1 ± 6.4	20.8 ± 6.3	−5.2	< 0.001
Psychological and behavioral	WHO-5 score	11.9 ± 4.7	16.8 ± 5.1	12.3 ± 4.9	13.4 ± 4.7	+3.8	< 0.001
Psychological and behavioral	MBI–emotional exhaustion	27.4 ± 7.2	22.6 ± 6.3	28.2 ± 7.5	27.9 ± 7.4	−4.5	< 0.001
Management performance	Average sick leave days	3.4 ± 1.8	2.1 ± 1.3	3.5 ± 2.0	3.2 ± 1.7	−1.0	< 0.001
Management performance	Average overtime hours	12.8 ± 5.4	10.0 ± 4.2	13.1 ± 6.1	12.5 ± 5.3	−2.2	0.001
Management performance	Absenteeism rate (%)	5.7 ± 2.1	3.2 ± 1.5	5.8 ± 2.2	5.5 ± 2.0	−2.2	< 0.001
Management performance	Work efficiency	112 ± 35	128 ± 38	110 ± 30	115 ± 32	+11	< 0.001

The OHMS intervention group and contemporaneous control group were matched at a 1:1 ratio using propensity score matching.

DID estimates were calculated as the change from baseline to the 6-month assessment in the OHMS intervention group minus the corresponding change in the matched contemporaneous control group.

Positive DID values indicate greater increases in the intervention group relative to controls, whereas negative DID values indicate greater decreases.

The subsequent 6-month monitoring period was not included in the primary effectiveness analysis.

OHMS, occupational health management system; DID, difference-in-differences; SPS-6, Stanford Presenteeism Scale; WHO-5, World Health Organization Five Wellbeing Index; MBI, Maslach Burnout Inventory.

For the primary outcome, SPS-6 scores decreased markedly in the intervention group from 20.8 ± 6.1 at baseline to 15.3 ± 5.2 at the 6-month assessment, whereas the matched control group showed only a minimal change from 21.1 ± 6.4 to 20.8 ± 6.3. The DID estimate for SPS-6 was −5.2 (*P* < 0.001), indicating a significant reduction in presenteeism attributable to the OHMS intervention.

For psychological wellbeing, WHO-5 scores increased from 11.9 ± 4.7 to 16.8 ± 5.1 in the intervention group, compared with a smaller increase from 12.3 ± 4.9 to 13.4 ± 4.7 in the matched control group. The DID estimate was +3.8 (*P* < 0.001), suggesting that OHMS implementation was associated with improved wellbeing. Emotional exhaustion, measured by the MBI, also decreased substantially in the intervention group from 27.4 ± 7.2 to 22.6 ± 6.3, whereas the control group remained largely unchanged. The DID estimate was −4.5 (*P* < 0.001).

Management and work performance indicators also improved after OHMS implementation. Compared with the matched control group, the intervention group showed greater reductions in average sick leave days, overtime hours, and absenteeism rate, as well as a greater increase in work efficiency. Specifically, average sick leave days decreased from 3.4 ± 1.8 to 2.1 ± 1.3 in the intervention group, compared with a decrease from 3.5 ± 2.0 to 3.2 ± 1.7 in the matched control group, yielding a DID estimate of −1.0 (*P* < 0.001). Average overtime hours decreased from 12.8 ± 5.4 to 10.0 ± 4.2 in the intervention group and from 13.1 ± 6.1 to 12.5 ± 5.3 in the matched control group, with a DID estimate of −2.2 (*P* = 0.001). Absenteeism rate decreased from 5.7 ± 2.1 to 3.2% ± 1.5% in the intervention group and from 5.8 ± 2.2 to 5.5% ± 2.0% in the matched control group, yielding a DID estimate of −2.2 (*P* < 0.001). Work efficiency increased from 112 ± 35 to 128 ± 38 in the intervention group, compared with an increase from 110 ± 30 to 115 ± 32 in the matched control group, with a DID estimate of +11 (*P* < 0.001).

As a supplementary robustness check, analyses using historical pre-implementation occupational health records showed directionally consistent patterns for SPS-6, WHO-5, MBI emotional exhaustion, absenteeism rate, and work efficiency, although these findings were considered exploratory and were not used as the primary basis for inference.

Overall, these findings indicate that the OHMS intervention significantly reduced presenteeism, improved psychological wellbeing, alleviated emotional exhaustion, and enhanced work-related performance indicators over the 6-month primary evaluation period. The DID estimates further support that these improvements were greater in the OHMS intervention group than in the matched contemporaneous control group, rather than reflecting simple within-group pre–post changes.

### 3.3 Associations between presenteeism and objective work performance indicators

Following the 6-month OHMS intervention, the intervention group exhibited significant improvements in presenteeism-related measures and objective work performance indicators. As shown in Table 4, SPS-6 scores decreased from baseline to the 6-month assessment, while WHO-5 scores increased and MBI emotional exhaustion scores decreased, indicating reduced presenteeism, improved psychological wellbeing, and alleviated emotional exhaustion.

**Table 4:** Baseline-to-6-month changes in presenteeism-related measures and their associations with changes in objective work performance indicators in the OHMS intervention group.

Indicator	Baseline, mean ±SD	6-month assessment, mean ±SD	Spearman's *r* with SPS-6 change	*P*-value
Medical error rate	0.32 ± 0.18	0.25 ± 0.15	−0.28	< 0.01
Outpatient record completion	250 ± 35	265 ± 38	0.32	< 0.01
Prescription processing time, min	12.5 ± 3.2	11.8 ± 2.9	−0.18	0.04
SPS-6 score	20.8 ± 6.1	15.3 ± 5.2	—	—
WHO-5 score	11.9 ± 4.7	16.8 ± 5.1	—	—
MBI emotional exhaustion	27.4 ± 7.2	22.6 ± 6.3	—	—

This table summarizes baseline-to-6-month changes within the OHMS intervention group.

Pre- and post-intervention values for SPS-6, WHO-5, and MBI emotional exhaustion are consistent with the intervention-group values reported in Table 3.

Spearman correlation coefficients were calculated between baseline-to-6-month changes in SPS-6 scores and baseline-to-6-month changes in each objective work performance indicator.

For indicators where lower values indicate improvement, including medical error rate and prescription processing time, changes were coded so that positive values represented improvement.

Therefore, a negative correlation between SPS-6 change and medical error rate improvement indicates that greater reductions in presenteeism were associated with greater improvements in medical error rates.

For SPS-6, WHO-5, and MBI emotional exhaustion, correlation coefficients are not repeated in this table because these variables serve as psychological outcome measures rather than objective performance indicators.

SPS-6, Stanford Presenteeism Scale; WHO-5, World Health Organization Five Wellbeing Index; MBI, Maslach Burnout Inventory; OHMS, occupational health management system.

In parallel, objective work performance indicators also improved. Medical error rates decreased from 0.32 ± 0.18 at baseline to 0.25 ± 0.15 at the 6-month assessment, outpatient record completion increased from 250 ± 35 to 265 ± 38, and prescription processing time decreased from 12.5 ± 3.2 min to 11.8 ± 2.9 min. These findings indicate that the OHMS intervention was associated not only with improved self-reported presenteeism and wellbeing but also with measurable improvements in work efficiency and medical quality.

Correlation analyses further supported the association between reductions in presenteeism and improvements in objective work performance. Spearman correlation coefficients were calculated between baseline-to-6-month changes in SPS-6 scores and corresponding changes in objective work performance indicators. For indicators where lower values indicate improvement, including medical error rate and prescription processing time, changes were coded so that positive values represented improvement. Greater reductions in SPS-6 scores were significantly associated with greater improvements in medical error rates, outpatient record completion, and prescription processing time. These findings suggest that reductions in presenteeism were accompanied by favorable changes in objective performance indicators.

Taken together, these results suggest that the OHMS intervention may help mitigate presenteeism among healthcare workers while improving work efficiency and medical quality. However, because these analyses were observational and based on within-group associations, the correlations between presenteeism-related changes and objective performance indicators should be interpreted as supportive rather than causal evidence.

### 3.4 Factors influencing presenteeism

**Figure 1:**
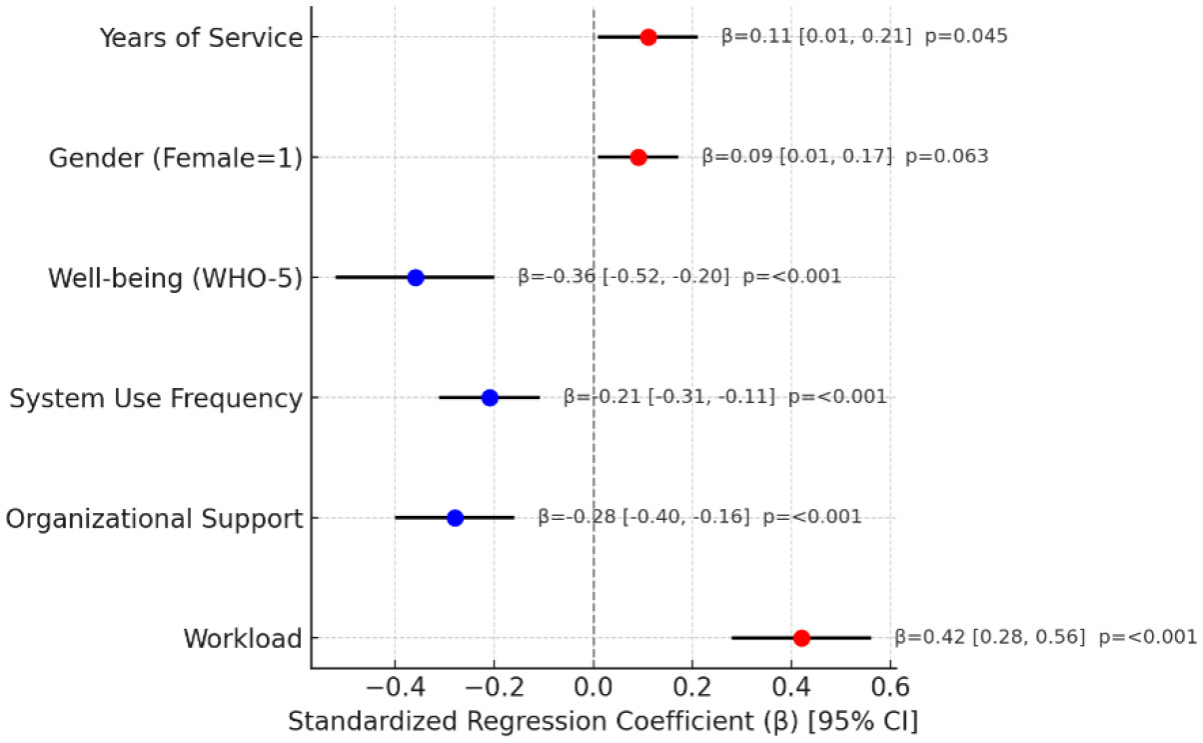
Standardized regression coefficients (β) with 95% confidence intervals for predictors of presenteeism.

Multiple linear regression analysis (Figure 1) demonstrated good overall model fit, with a high explanatory power (*R*^2^ = 0.62, adjusted *R*^2^ = 0.59, *P* < 0.001).

After controlling for gender and years of service, workload, organizational support, system usage frequency, and wellbeing (WHO-5 score) were all significant predictors of healthcare worker presenteeism. Workload had a significant positive effect on presenteeism (β = 0.42, *P* < 0.001), indicating that higher work demands increase the risk of working while unwell. Organizational support (β = −0.28, *P* < 0.001) and system usage frequency (β = −0.21, *P* < 0.001) were significant negative predictors, suggesting that management support and systematic interventions can effectively reduce presenteeism. Wellbeing exhibited the strongest negative effect (β = −0.36, *P* < 0.001), indicating that higher psychological health is associated with lower presenteeism. Years of service had a modest positive effect (β = 0.11, *P* = 0.045), suggesting slightly higher risk among more experienced staff. Gender differences were not statistically significant (*P* = 0.063) (29).

### 3.5 Structural equation modeling (SEM) analysis

The hypothesized model, based on the Job Demands–Resources (JD–*R*) theoretical framework, was tested using structural equation modeling (SEM). In the model, workload was specified as the exogenous variable, wellbeing as the mediator, and presenteeism as the outcome variable, with organizational support and system use frequency included as moderating variables.

**Figure 2:**
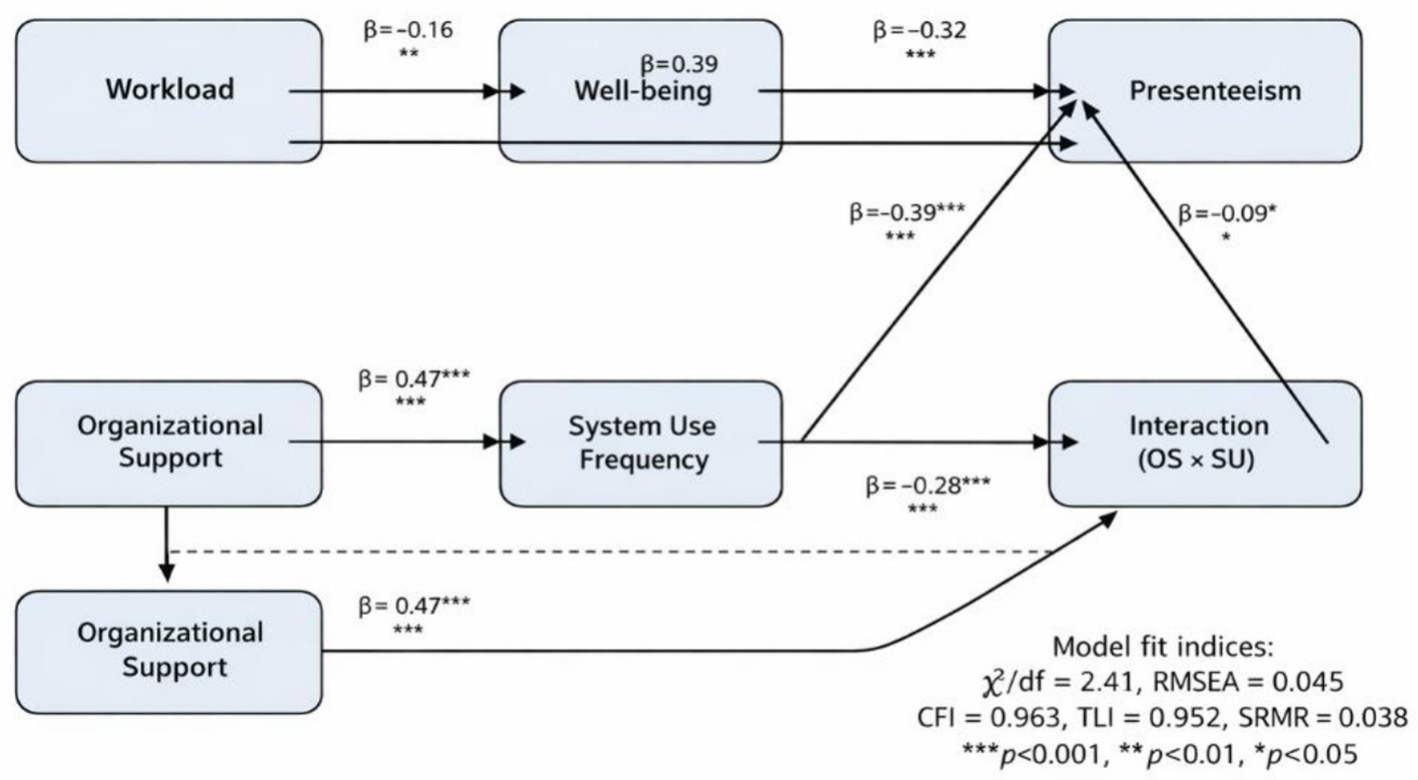
Standardized path diagram of the structural equation model (β values and significance levels).

Model fit indices indicated good fit (χ^2^/df = 2.41, RMSEA = 0.045, CFI = 0.963, TLI = 0.952, SRMR = 0.038), suggesting adequate statistical adequacy. Standardized path coefficients are presented in Table 5, and the visualized structural model is shown in Figure 2.

**Table 5:** SEM path estimates (standardized coefficients).

Path	Standardized coefficient (β)	Standard error (SE)	t-value	*P*-value	95% CI (bootstrap, *n* = 5,000)	Effect type
Workload → presenteeism	0.39	0.07	5.42	< 0.001	(0.27, 0.51)	Direct effect
Workload → wellbeing	−0.16	0.05	−3.20	0.001	(−0.25, −0.07)	Antecedent of mediation
Wellbeing → presenteeism	−0.32	0.08	−4.00	< 0.001	(−0.46, −0.18)	Mediation path
workload → wellbeing → presenteeism	0.16	0.05	3.10	0.002	(0.06, 0.26)	Indirect effect (partial mediation)
Organizational support → system use frequency	0.47	0.06	6.83	< 0.001	(0.35, 0.58)	Antecedent of moderation
System use frequency → presenteeism	−0.28	0.07	−3.99	< 0.001	(−0.39, −0.17)	Moderation path
Organizational support × system use frequency → presenteeism	−0.09	0.04	−2.25	0.024	(−0.17, −0.01)	Interaction/moderation effect

β: Standardized path coefficient.95% CI was calculated using bootstrap resampling with 5,000 iterations.

Model fit indices indicate good overall fit: χ^2^/df = 2.41, RMSEA = 0.045, CFI = 0.963, TLI = 0.952, SRMR = 0.038.

Results showed that workload had a significant negative effect on wellbeing (β = −0.16, *p* < 0.01), and wellbeing significantly negatively predicted presenteeism (β = −0.32, *p* < 0.001), indicating a partial mediating role of wellbeing. Workload also had a significant positive direct effect on presenteeism (β = 0.39, *p* < 0.001), suggesting that high work demands directly increase the risk of working while unwell.

Organizational support positively influenced system use frequency (β = 0.47, *p* < 0.001), and system use frequency negatively predicted presenteeism (β = −0.28, *p* < 0.001). Moreover, the interaction term (Organizational Support × System Use Frequency) had a modest negative moderating effect on presenteeism (β = −0.09, *p* < 0.05; Table 5).

### 3.6 Correlation between subjective presenteeism and objective work performance

To validate the construct of the SPS-6 scale, baseline data from all participants were analyzed. As shown in Table 6, SPS-6 total scores were positively correlated with average prescription processing time (*r* = 0.32, *p* < 0.01) and negatively correlated with monthly outpatient record completion (*r* = −0.28, *p* < 0.01). Additionally, participants with higher SPS-6 scores exhibited relatively higher near-miss reporting rates (*r* = 0.25, *p* < 0.05).

**Table 6:** Spearman correlations between baseline SPS-6 scores and objective work performance indicators (*n* = 300).

Objective performance indicator	Spearman's *r*	*P*-value
Average prescription processing time	0.32	< 0.01
Monthly outpatient record completion	−0.28	< 0.01
Near-miss reporting rate	0.25	< 0.05

Although these correlations were of moderate strength, the directions were consistent with theoretical expectations, providing objective support for the criterion-related validity of the SPS-6 scale in this study population. Post-intervention, as SPS-6 scores significantly decreased, average prescription processing time also shortened significantly (*p* < 0.05), indicating that the intervention not only improved subjective perceptions but also enhanced objective work efficiency.

### 3.7 Qualitative analysis results

Semi-structured interviews were conducted with 30 healthcare workers across different risk levels, yielding three core themes:

Professional responsibility and self-sacrifice culture: most participants reported beliefs such as “minor illness does not need reporting” or “if it does not affect work, it is considered healthy,” reflecting the suppressive effect of traditional professional ethics on health risk perception.

Behavioral guidance effect of system feedback: participants generally perceived that OHMS-provided “health reminders,” “point-based incentives,” and “risk level feedback” enhanced self-awareness and health responsibility. Some actively sought psychological counseling or adjusted their daily routines in response.

Importance of organizational support and team culture: effective peer support and department-level health education significantly increased adherence to system use, shifting health management from an individual behavior to a collective consensus.

These qualitative findings were consistent with quantitative results, further confirming the role of the occupational health management system in behavior reinforcement and cultural transformation within healthcare settings.

## 4 Discussion

This study, grounded in the Job Demands–Resources (JD–*R*) theory and Health Belief Model (HBM), developed and validated a structural model of presenteeism among healthcare workers and evaluated the effectiveness of a digital Occupational Health Management System (OHMS) (13, 14, 16, 19). The findings demonstrated that workload significantly increased presenteeism both directly and indirectly through reduced wellbeing, while organizational support and system usage frequency exerted protective effects. The structural equation model showed good fit (χ^2^/df = 2.41, RMSEA = 0.045, CFI = 0.963), supporting the hypothesized “organizational support–system use–wellbeing–presenteeism” pathway.

The intervention led to significant improvements in both subjective and objective outcomes. SPS-6 scores decreased, WHO-5 scores increased, and multiple performance indicators—including sick leave days, overtime hours, medical error rates, and outpatient record completion—showed favorable changes (23, 24). These findings indicate that the OHMS intervention not only reduced perceived presenteeism but also translated into measurable improvements in work efficiency and healthcare quality.

Mechanistically, the results support the JD–*R* framework, in which excessive job demands deplete individual resources and impair functioning. In this study, workload negatively affected wellbeing, which in turn increased presenteeism, confirming a partial mediation effect. This finding aligns with the “resource depletion → functional impairment” pathway and highlights wellbeing as a central mechanism linking workload to work performance. In parallel, organizational support indirectly reduced presenteeism by promoting system usage, consistent with the resource compensation hypothesis (13, 14).

Notably, system usage frequency played a key role in reinforcing health-related behaviors. Frequent interaction with OHMS enhanced risk awareness, promoted self-regulation, and facilitated access to intervention resources. The interaction analysis further suggested that high organizational support combined with frequent system use produced stronger reductions in presenteeism, particularly in high-stress clinical environments. These findings indicate that digital health systems can act as behavioral amplifiers when embedded within supportive organizational contexts.

Stratified analyses revealed heterogeneous intervention effects across professional groups. Nurses exhibited the largest reduction in presenteeism and the greatest improvement in wellbeing, likely due to higher baseline workload and emotional labor. Physicians also demonstrated substantial improvements, particularly in work efficiency, although workload remained a persistent risk factor. In contrast, medical and administrative staff showed more moderate changes, suggesting that baseline workload intensity and role characteristics influence intervention responsiveness. These findings underscore the need for role-specific intervention strategies.

The integration of subjective and objective indicators represents a notable strength of this study. The observed correlations between SPS-6 scores and objective performance metrics, including medical error rates and outpatient record completion, provide empirical support for the validity of presenteeism measurement. The consistency between subjective improvement and objective performance gains further strengthens the evidence for the effectiveness of the OHMS intervention.

Potential bias arising from non-active users should also be considered. Non-active participants were more frequently observed in administrative and logistical departments, and digital literacy was positively associated with system engagement. Regression analysis indicated that non-active status had a modest effect on outcome measures, suggesting that differential system usage may partially influence the estimated intervention effects. This highlights the importance of improving user engagement and accessibility in future implementations.

From a practical perspective, this study provides empirical support for a scalable digital occupational health management model (28). By integrating real-time monitoring, risk stratification, and personalized intervention, OHMS enables a transition from passive health management to proactive and dynamic risk control. Embedding such systems into hospital performance frameworks may facilitate the dual optimization of workforce wellbeing and organizational efficiency.

Several limitations should be acknowledged. First, the quasi-experimental design may be subject to residual confounding despite the use of matching techniques. Second, the study was conducted in a single center, which may limit generalizability. Third, the follow-up period was relatively short, and long-term sustainability of intervention effects remains to be established. Fourth, the difference-in-differences (DID) analysis assumes that, in the absence of intervention, the intervention and control groups would have followed parallel outcome trends. Due to the lack of multiple pre-intervention measurements, this assumption could not be formally verified. Although propensity score matching and baseline outcome equivalence improve the plausibility of DID estimates, unobserved time-varying confounders may still bias the results. Therefore, the reported DID effects should be interpreted as quasi-causal rather than strictly causal. Future studies incorporating multiple pre-intervention time points or randomized designs are warranted to confirm the causal impact of OHMS interventions.

Future research should extend follow-up duration to evaluate long-term effects and behavioral sustainability. In addition, multi-center studies are needed to validate the generalizability of the OHMS model across different healthcare settings. Further integration of qualitative and quantitative approaches may also provide deeper insights into behavioral mechanisms and facilitate optimization of digital health interventions.

In conclusion, this study demonstrates that a digital occupational health management system can effectively reduce presenteeism among healthcare workers by enhancing wellbeing and organizational support. The findings provide a practical framework for implementing data-driven occupational health strategies and highlight the importance of integrating technological, organizational, and behavioral approaches to improve healthcare workforce outcomes.

## Data Availability

The raw data supporting the conclusions of this article will be made available by the authors, without undue reservation.
