# Supplementary material for: Quasi-experimental evaluation of a digital occupational health management system on presenteeism and work efficiency among healthcare workers
Source: Front Public Health. 2026 Jun 4;14:1827192. doi: 10.3389/fpubh.2026.1827192 (PMC13275680; doi:10.3389/fpubh.2026.1827192)
Supplement: Supplementary file 1 [file Table_1.docx]

**Supplementary Table S1. Baseline characteristics of the OHMS intervention group and eligible contemporaneous control pool before and after propensity score matching**

| **Variable** | **Intervention group before PSM (n = 300)** | **Eligible contemporaneous control pool before PSM (n = 420)** | **SMD before PSM** | **Intervention group after PSM (n = 300)** | **Matched contemporaneous control group after PSM (n = 300)** | **SMD after PSM** |
| --- | --- | --- | --- | --- | --- | --- |
| Age, years | 35.2 ± 6.4 | 35.8 ± 6.7 | 0.09 | 35.2 ± 6.4 | 35.4 ± 6.5 | 0.03 |
| Female, % | 68.7 | 70.0 | 0.03 | 68.7 | 69.3 | 0.01 |
| Years of service | 9.7 ± 5.3 | 10.1 ± 5.5 | 0.07 | 9.7 ± 5.3 | 9.8 ± 5.4 | 0.02 |
| Physicians, % | 32.7 | 31.3 | 0.03 | 32.7 | 32.0 | 0.02 |
| Nurses, % | 52.0 | 53.7 | 0.03 | 52.0 | 52.3 | 0.01 |
| Medical technicians/administrative staff, % | 15.3 | 15.0 | 0.01 | 15.3 | 15.7 | 0.01 |
| Baseline SPS-6 score | 20.8 ± 6.1 | 21.1 ± 6.4 | 0.05 | 20.8 ± 6.1 | 21.1 ± 6.4 | 0.05 |
| Baseline WHO-5 score | 11.9 ± 4.7 | 12.3 ± 4.9 | 0.08 | 11.9 ± 4.7 | 12.3 ± 4.9 | 0.08 |
| Baseline MBI emotional exhaustion | 27.4 ± 7.2 | 28.2 ± 7.5 | 0.11 | 27.4 ± 7.2 | 27.9 ± 7.4 | 0.07 |
| Average weekly working hours | 50.1 ± 7.3 | 51.4 ± 7.6 | 0.17 | 50.1 ± 7.3 | 50.6 ± 7.4 | 0.07 |
| Night shifts per month | 5.6 ± 3.5 | 6.1 ± 3.7 | 0.14 | 5.6 ± 3.5 | 5.8 ± 3.6 | 0.06 |

**Note:** PSM, propensity score matching; SMD, standardized mean difference; OHMS, Occupational Health Management System; SPS-6, Stanford Presenteeism Scale; WHO-5, World Health Organization Five Well-Being Index; MBI, Maslach Burnout Inventory. Propensity scores were estimated using age, gender, years of service, and department type. The OHMS intervention group was fixed at n = 300. A total of 420 eligible contemporaneous non-intervention controls were available before matching, and 300 matched controls were selected from this eligible control pool using 1:1 nearest-neighbor matching without replacement, with a caliper width of 0.2 of the standard deviation of the logit of the propensity score. An SMD < 0.10 was considered indicative of acceptable covariate balance. Continuous variables are presented as mean ± standard deviation, and categorical variables are presented as percentages.
